# Engineered kin recognition specificities in the TraA cell surface receptor

**DOI:** 10.1093/ismejo/wrag102

**Published:** 2026-04-23

**Authors:** Tingting Guo, Daniel Wall

**Affiliations:** Department of Molecular Biology, University of Wyoming, Laramie, WY 82071, United States; Department of Molecular Biology, University of Wyoming, Laramie, WY 82071, United States

**Keywords:** *Myxococcus xanthus*, cell surface receptor, outer membrane exchange, kin discrimination, greenbeard gene

## Abstract

Recognizing self versus nonself is a crucial step in the development of multicellularity. The social bacterium *Myxococcus xanthus* is a tractable model organism for studying this transition from single-cell to multicellular life. The polymorphic cell-surface receptor TraA directs cooperative behaviors toward kin. TraA is a highly specific receptor, capable of recognizing other TraA proteins with identical or nearly identical sequences by homotypic binding, but the molecular basis of this specificity remains poorly understood. Here, we generated a targeted TraA mutant library comprising thousands of variants with substitutions at 10 predicted specificity-determining residues. Screening revealed variants with altered recognition profiles, often resulting in promiscuous and/or heterotypic TraA–TraA interactions. We further identified key residues that govern specificity, as substitutions at these positions rewired recognition outcomes. Finally, we propose an evolutionary model in which new TraA specificities arise through promiscuous intermediate states shaped by reward-punishment dynamics. Together, these findings demonstrate the malleability of TraA specificity and provide molecular and evolutionary insight into social recognition.

## Introduction

Cellular self-recognition is essential for the organization, integrity, and function of multicellular organisms because it allows individual cells to distinguish self from nonself and beneficial neighbors from harmful ones. Through specific cell–cell recognition systems, cells can selectively adhere, communicate, and coordinate behaviors such as migration, differentiation, and programmed cell death, ensuring proper tissue assembly [1]. Self-recognition mechanisms also prevent detrimental interactions with pathogens or inappropriate cell mixing or fusion. During development, these systems enable processes like cell sorting, pattern formation, and neural wiring, while in adult organisms they support tissue maintenance, regeneration, and immune surveillance [2, 3]. Together, cellular self-recognition provides a molecular framework that allows complex multicellular life to function as an integrated, cooperative whole rather than as a collection of independent cells.

A model organism to study cellular self-recognition is the motile soil bacterium *Myxococcus xanthus*. It is renowned for its complex social behaviors where its lifestyle involves transitions from solitary cells to cooperative tissue-like multicellular structures, which involves clonal or self-recognition assessment during aggregation and development [4]. This recognition of self allows cooperative behaviors to be directed toward clonemates or close kin. To achieve such multicellular genomic uniformity, cells rely on kin recognition and discrimination systems. Kin recognition refers to genetic determinants that identify clonemates or close kin (siblings with a recent common ancestor), whereas kin discrimination involves antagonism against individuals that are not clonal (nonkin) with respect to their social genes [5, 6]. The best-studied kin recognition system in myxobacteria is TraAB-mediated outer membrane exchange (OME), which enables *M. xanthus* to distinguish between self and nonself (nonkin). During OME, cells rapidly exchange substantial amounts of outer membrane (OM) proteins, lipids, and lipopolysaccharides through direct cell–cell contacts [7–9].

OME governs diametrically opposed functions, fostering both cooperation and antagonism among related cells. For cooperation, OME promotes shared cellular resources with clonemates. For instance, OME was initially discovered for its ability to rescue defects in a subset of gliding motility mutants through extracellular complementation, where wild-type (WT) motility proteins from donor cells are transferred to mutants lacking those proteins [9, 10]. OME also rejuvenates damaged cells with compromised membranes and can transfer adaptation phenotypes by exchanging beneficial components [11, 12]. Finally, when the TraAB receptors are overexpressed, cells adhere to one another and their motility is altered leading to emergent population behaviors [13]. In contrast, OME also mediates antagonism, defined as kin discrimination, by exchanging lipoprotein toxins between nonclonal cells that lack a cognate suite of immunity proteins, which are not transferred [14–16].

Sharing of cellular content is an intimate process mediated by cell–cell recognition determined by TraA–TraA homotypic binding between cell surface receptors. This bidirectional exchange requires direct contact between cells expressing identical or nearly identical TraA receptors. TraA is highly polymorphic, and its variable domain (VD) specifies recognition specificity by homotypic binding [17]. TraA functions with its operonic partner, TraB, which is essential for OME but does not influence specificity [18]. Because lipids are exchanged along with proteins, OME is thought to occur by transient OM fusion, with TraAB serving as fusogens [19]. Among the many different proteins transferred by OME are a suite of polymorphic lipoprotein toxins. There are six discrete SitA toxin families, with each family typically represented multiple times in genomes adjacent to their cognate SitI immunity proteins. SitA (swarm inhibition toxin) is named for its ability to inhibit outward swarming. This occurs when mixed cultures contain compatible TraA receptors, but divergent *sitAI* loci, are placed on agar, resulting in OME-mediated cell poisoning and, consequently, a block in motility and colony expansion [9, 14]. Altogether, strains typically contain over 30 *sitAI* loci and up to 83, resulting in an astronomical degree of discrimination specificity between strains expressing compatible TraA receptors but divergent *sitAI* loci [16]. This interplay between OME and toxin transfer likely drives the emergence and maintenance of TraA polymorphisms, and hence recognition specificity seen in nature, because OME between different strains with compatible TraA receptors is lethal. Additionally, SitA can transfer serially between cells, thus acting like an infectious agent that disseminates through populations mediated by OME. Accordingly, horizontal transfer of *sitAI* loci likely contributes to the diversification of myxobacterial social groups [20].

Myxobacteria form distinct recognition groups based on their TraA receptors. Swapping the VD between *traA* alleles can reprogram recognition specificity. Furthermore, even substituting a single amino acid residue—A/P205—within the VD can alter specificity [21]. To date, experimental studies have identified 10 TraA recognition groups, designated A through J, and comparative sequence analysis indicates that many more exist in nature [18]. Despite TraA’s key role in the social interactions of myxobacteria, significant gaps remain in our understanding of the molecular basis of recognition specificity.

We hypothesized that OME originally evolved as a cooperative behavior to facilitate the transition to multicellularity, which subsequently evolved as an antagonism platform. Clues about these paradoxical roles are gleaned from genomes. Here, *sitAI* loci are frequently found on mobile selfish elements, including prophage, transposons, and plasmids [15, 20]. In turn, these toxin-immunity cassettes provide a selective advantage for elements to expand and be maintained in populations because TraA-compatible siblings that lack them are killed. Comparative genomic studies from sympatric isolates indeed found that these elements are rapidly disseminated by horizontal gene transfer (HGT) in populations [15]. Thus, OME provides cooperative benefits to clonemates and discriminatory functions against nonclonemates that happen to have a compatible TraA receptor but different SitA toxins. Therefore, OME-based kin discrimination is driven by selfish elements, which also provide a selective basis for TraA polymorphisms and hence recognition specificity.

Protein–protein specificity is primarily determined by a subset of residues that strongly covary [22]. These critical residues are typically located at the interface surface between interacting proteins [23, 24]. Here, to investigate the molecular basis of TraA−TraA specificity, we used sequence alignments, AlphaFold modeling, and experimental data to identify candidate TraA residues involved in recognition [18, 21, 25]. To systematically explore these residues, we created a large, targeted combinatorial library of TraA variants and screened them for altered specificities. Our results reveal that changing a small subset of residues within the VD is sufficient to reprogram specificity. We found that many of these variants exhibited promiscuous binding, capable of recognizing multiple TraA groups. However, most of them could not recognize self; thus, these variant alleles switched from homotypic to heterotypic allele recognition—suggesting the emergence of novel homotypic recognition alleles is rare. These findings further imply that evolved TraA receptors likely evolve through intermediate promiscuous states. We propose that these intermediates are then selected against, as SitA toxin exchange penalizes heterotypic and/or promiscuous *traA* alleles. Collectively, this work advances our understanding of the molecular and evolutionary basis of social recognition and diversity within the Myxococcota phylum and has implications for other allotype recognition systems [26–28].

## Materials and methods

### Bacterial strains and growth conditions

Table S1 lists plasmids and strains used in this study. *Myxococcus xanthus* strains were cultured in the dark at 33°C with shaking in CTT media (1% [w/v] casitone, 1 mM KH_2_PO_4_, 8 mM MgSO_4_, 10 mM Tris–HCl, pH 7.6). For ½ CTT, casitone was reduced to 0.5%. *Escherichia coli* cultures were grown in Luria broth (LB) at 37°C. For solid media, agar was added at 1.5% or 0.5% (w/v). Antibiotics were added at the following concentrations when indicated: 50 μg/ml kanamycin (Km) for *M. xanthus* and *E. coli*, 15 μg/ml oxytetracycline (oTc) for *M. xanthus*, and 10 μg/ml tetracycline (Tc) for *E. coli.* TPM buffer (CTT without casitone) was used to wash cells.

### Plasmid and strain construction

Primers used in this study are listed in Table S2. The *traA^F-P205A^* library was cloned into pMR3487 [29] with the *traB* gene and expression was driven by an inducible P_IPTG_ promoter. To create the plasmid backbone for library construction, the *traAB^Mf^* genes from pPC4 were amplified by PCR. The resulting fragment was then subcloned into pMR3487 (linearized with XbaI and KpnI) with Gibson Assembly Master Mix (New England Biolabs).

The TraA-G^*^ (DDPGA → LN) variant was generated by deleting codons 69–73 from plasmid pPC26 and inserting the codons CTC and AAC coding for leucine and asparagine, respectively. Primers with substituted bases were used for PCR amplification. The resulting fragments were assembled using Gibson Assembly.

To generate TraA-A^*^ (insertion of DDP before AV) plasmid, primers used for PCR amplification were engineered with codons GAT, GAC, and CCG coding for aspartic acid, aspartic acid, and proline, respectively. The resulting fragments were cloned via Gibson Assembly.

A markerless in-frame deletion of *cglC* was constructed in strain DK1253 (*tgl1*). Briefly, pΔ*cglC*, containing a *cglC* deletion cassette and a Km^R^-*galK* selection/counter-selection cassette, was electroporated into DK1253. Homologous recombinants were first selected by Km^R^, and then plasmid excision was counter-selected on 3% galactose plates. This markerless deletion of *cglC* generated strain DW2929, which was confirmed by PCR with flanking primers and phenotype analysis. To create markerless in-frame deletion of *traA* in DW2929, pDP28 containing a *traA* deletion cassette was transformed and selected as described above. The resulting markerless deletion of *traA* generated strain DW2930.

All plasmids were verified by PCR, restriction enzyme digestion, and DNA sequencing. Verified plasmids were then electroporated into *M. xanthus* cells and selected with appropriate antibiotics.

### Library construction and screening

To identify candidate residues involved in TraA−TraA recognition, we used AlphaFold2 to predict surface residues participating in noncovalent interactions between TraA monomers. This was conducted with different TraA alleles to develop a consensus of homophilic binding residues. This analysis identified 10 predicted contact residues that were selected for mutagenesis in library construction, along with a control surface residue predicted to be outside of the binding interface. Amino acid substitutions at each site were based on naturally occurring residues found in TraA orthologs. Specifically, sequence alignments of 57 TraA orthologs from recognition groups B to F, which contain no indels among themselves, were used to select substitution residues.

Plasmid pMR3487-*traAB^Mf^* (aka pTG2909) served as the template for library construction and validation by Twist Biosciences using their proprietary methods. This library DNA was transformed and amplified in *E. coli* DH5α. The resulting transformants were pooled, and plasmid DNA was purified, dialyzed, and electroporated into *M. xanthus* strains, resulting in homologous recombination at an ectopic site on chromosome [29], distant from the deleted *traAB* locus, by selecting oTc^R^.

For library screening, the stimulation assay was used as essentially described [18]. Briefly, nonmotile and nonstimulatable donor strains were mixed with nonmotile stimulatable recipient strains (Δ*cglC tgl1*) at a 1:1 ratio, and placed onto ½ CTT agar with 2 mM CaCl_2_ for ~48 hr at 33°C. For library screens, mid-exponential overnight *M. xanthus* cultures were mixed with a donor strain(s) containing a specific *traA* allele and a recipient library as outlined. Recipients that contained compatible TraA receptors with the given donor resulted in transient rescue of motility by OME that was manifested as flares of cells emerging from the inoculum on agar plates. Isolated colonies from such flares were obtained for further analysis. To document stimulation, the edges of colonies were imaged with Nikon E800 phase contrast microscope with a 10× objective lens coupled to a digital imaging system.

### Phylogenetic analysis

Maximum likelihood (ML) phylogenetic trees were constructed in PhyML 3.0 [30]. We first identified the best-fit models based on the Akaike Information Criterion (AIC) in ProTest 3 [31]. For the analysis of 57 TraA VD orthologs from the *Myxococcaceae* family, the best fitting model (Q.insect+R + F) selected by ProtTest was used, and 1000 bootstrap replicates were performed. In addition, sequences of the 193 TraA VDs spanning of *Cystobacterineae* suborder were used to generate an ML tree. For this analysis, the model (Q.yeast+R + F) was selected, and 1000 bootstrap replicates were performed. Phylogenetic trees were visualized with iTOL [32].

### Cell–cell adhesion assay

Strains and cocultures were mixed as indicated and incubated overnight with shaking at 300 rpm at 33°C. Cultures were grown to mid-log phase at a density of ~1 × 10^7^ cells per ml. Cell suspensions were directly mounted on glass slides and examined using a Nikon E800 microscope equipped with a 60× oil objective lens and FITC or Texas Red filter sets coupled to an imaging system.

### Protein transfer assay

Transfer assays were done as described [33]. In brief, to test SS_OM_-mCherry or SS_OM_-GFP transfer, strains were grown to mid-log phase (~5 × 10^8^ cells per ml) and mixed as indicated and spotted on ½ CTT 1.2% agar pads. Images taken after spots dried on the agar pads or incubated at 33°C for indicated times.

## Results

### TraA specificity-determining residues

**Figure 1:**
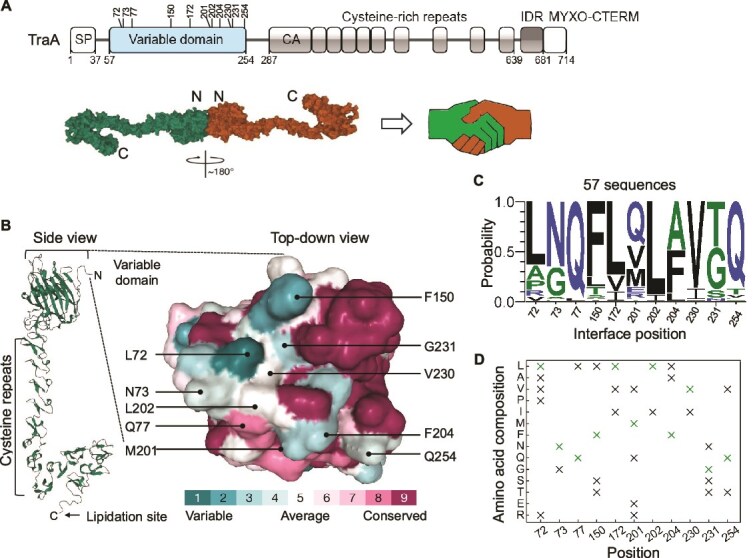
Library rationale. (A) Domain organization of TraA (group F, *M. fulvus* HW-1 receptor) with VD residues targeted for substitution (top), and AlphaFold predicted TraA homodimer, where monomers are rotated approximately 180° with respect to each other, like a handshake. IDR, intrinsically disordered region. (B) Side and top-down view of the VD, where residues degree of conservation is color-coded based on a cohort of 57 sequences. (C) WebLogo of 10 test positions and one control position (residue 172) targeted for substitutions based on 57 TraA orthologs from groups B–F. Residues designated by hydrophobicity: hydrophilic (blue), neutral (green), and hydrophobic (black). (D) Shows 24 possible substitutions at 11 positions in library design. Parent HW-1 sequences in green.

TraA contains a VD, cysteine-rich repeats, and a C-terminal protein sorting tag called MYXO-CTERM (Fig. 1A) [34, 35]. We previously showed that TraA−TraA recognition is highly selective, with specificity determined by VD polymorphisms [17]. To understand the molecular basis of recognition, we used AlphaFold2 to predict surface residues involved in interfacial interactions where noncovalent bonds between monomers were visualized in RCSB PDB (Figs S1 and S2) [25, 36]. AlphaFold predicted a TraA−TraA homodimer in a “head-to-head” configuration, formed by approximately a 180° rotation between monomers. The interface residues reside in the VD, and the orientation of homodimer monomers was analogous to a “handshake” (Figs 1A and S1). This head-to-head configuration was consistent with our prior work that the VD governs cell–cell recognition and provides a structural basis for understanding TraA specificity. We then generated a number of AlphaFold2 homodimer structures, which included representatives from six of our experimentally determined TraA recognition groups, i.e. A–F (Fig. S2) [17, 18]. This analysis identified 10 surface positions that were consistently found at the interface between monomers and were frequently involved in noncovalent TraA−TraA bonds (Figs 1A, 1B, and S2). These interfacial residues also exhibited sequence variability and, consequently, there were differences in the predicted roles particular positions played in homodimer interactions (Fig. S2). Nevertheless, we identified 10 plausible positions involved in TraA−TraA recognition specificity. Since our structural analysis focused are relatively small cohort of TraA recognition groups, these 10 positions were not comprehensive, as more divergent *traA* alleles contain numerous indels and SNPs not pursued here.

We generated sequence alignments to identify amino acids naturally found at these 10 interfacial positions. To do so, we restricted the alignment to 57 sequences that contained no VD indels, which consisted of the experimentally validated recognitions groups B–F [21]. This alignment revealed varying degrees of sequence variation (Figs 1C and S3).

### Library construction

Experimentally, we sought to interrogate the role of these 10 positions in specificity. Specifically, we sought to identify TraA variants that (i) altered their specificity from one recognition group to another, (ii) were promiscuous, recognizing multiple recognition groups, (iii) engaged in heterotypic binding, failing to recognize self, and (iv) enable a detailed structural understanding of specificity to facilitate rational design of new TraA recognition groups.

For library design, we selected a group F allele (*M. fulvus* HW-1) where amino acid substitutions were based on residues found in our alignment (Figs 1C and S3). This strategy limits library complexity by using naturally occurring amino acids found at those positions. As an internal control, we included a variable noninterfacial surface residue, designated as position 5 (aka 172) (Fig. 1C). Each of these 11 positions included 2 to 5 amino acid substitutions in the library, resulting in a total of 24 substitutions across the selected positions (Fig. 1D). Furthermore, in the group F parent allele, we changed the endogenous P205 residue to A205, which abolished parent allele recognition (below). This targeted synthetic *traA^F-P205A^* library was constructed by Twist Biosciences, with a theoretical diversity of 172,800 variants, where each clone, on average, contained five substitutions. After amplification and purification from *E. coli*, suicide plasmids were transformed into *M. xanthus.*

### Screen sensitivity and library assessment

To identify variant *traA* alleles with altered specificity, we employed a “stimulation assay” [10, 18]. This assay was based on the ability of donor cells to transfer missing motility proteins to recipient cells by OME, thus transiently restoring gliding motility. To test the sensitivity in our screen, we mixed donor and recipient strains at various ratios. Both strains were nonmotile and shared the same *traA* allele, where the *cglC^−^ tgl^−^* recipient was rescued by OME transfer of the CglC and Tgl proteins from the donor, thus restoring A- and S-motility (Fig. S4A) [4]. Strikingly, rare positive recipients with restored motility were detected at donor:recipient ratios as low as 1:200 000 cells (Fig. S4B), where a nonmotile/nonstimulable control strain was included to mimic nonfunctional library clones. We conclude that the stimulation assay was exquisitely sensitive and could detect rare *traA* clones with altered specificities.

**Figure 2:**
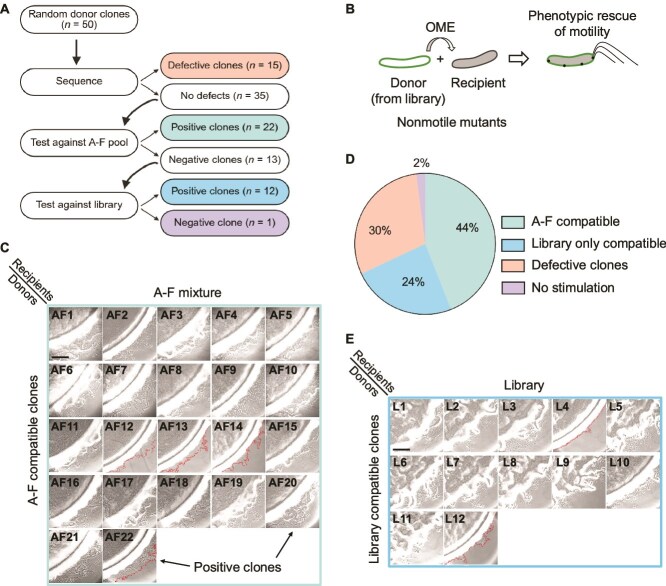
TraA library characterization from stimulation assays. (A) Flowchart of characterization steps of 50 random *traA* donor clones. Clones tested against a pool of recipients belonging to recognition groups A–F or a *traA* library pool. *n* refers to the number of clones identified at each step. (B) Illustration of how donor cells transfer missing motility proteins to recipient strain by OME in a stimulation assay. (C) Stimulation results for donor positive clones among a pool of recipient strains expressing TraA recognition groups A–F. Dotted red lines mark swarm fronts for clones exhibiting weak stimulation. (D) Activity distribution among 50 random clones. (E) As in C, except recipient strain pool from *traA^F-P205A^* library. Scale bar, 200 μm.

To assess library quality and screen feasibility, we transformed a pilot library into a nonmotile Δ*traA* donor (DW2302) and randomly picked 50 clones for characterization. Of these, 15 (30%) contained unintended *traA* mutations that were excluded from further analysis (Fig. 2A). Among the remaining donor clones, 22 (44%) recognized one or more members from a recipient pool consisting of TraA A–F recognition groups (Fig. 2A–D). The remaining 12 clones exhibited unique recognition patterns, as they did not recognize groups A–F, but instead recognized various alleles within a recipient (DW2930) *traA^F-P205A^* library pool (Fig. 2E). Finally, one donor clone was not active against the recipient library. Together, these results indicated our library contained many *traA* alleles with altered or novel specificities.

### TraA variants with altered recognitions

**Figure 3:**
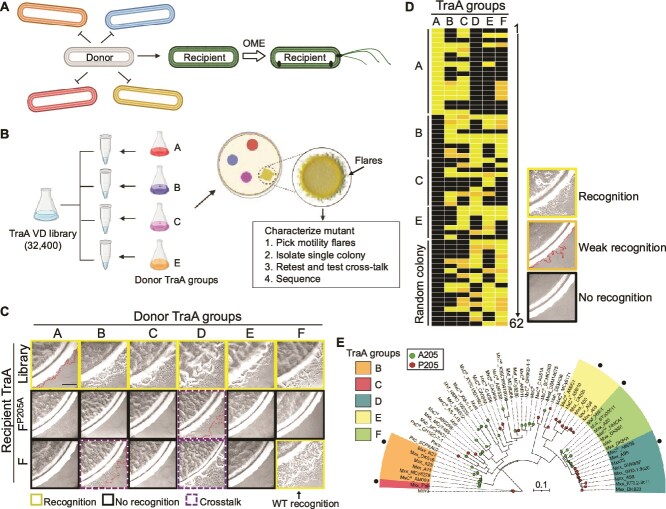
TraA library screen and recognition profiles. (A) Schematic of library screen. Only library recipients (colored cells) with a compatible TraA receptor (arrow) to a given donor receptor (gray) will engage in OME to transfer proteins that rescue motility defect. (B) Overview of the screening strategy against TraA recognition groups A, B, C, and E. (C) Stimulation tests of library compatibility against TraA groups A–F. As controls, the original TraA*^F^* receptor and the TraA*^F-P205A^* substituted receptor backbone used for library construction are shown. Scale bar, 200 μm. (D) Summary of 62 positive library clones tested against TraA groups A–F. Clones are sequentially numbered 1 through 62. (E) ML tree based on 57 VDs of Myxococcaceae TraA orthologs used for library design (Fig. 1). Each allele prefix shows the taxonomic origin (Table S3). Only TraA groups B–F sequences are shown because they contain no indels among themselves. Experimentally determined recognition groups are indicated. Black dots indicate the corresponding strain used in panels C and D. Scale bar, number of substitutions per residue.

To identify variants with altered recognitions, the *traA^F-P205A^* library was transformed and integrated into the chromosome of the DW2930 recipient strain (Δ*traA* Δ*cglC tgl1*). This pooled library of approximately 32,400 transformants was screened against four donor strains expressing naturally occurring TraA recognition groups—A, B, C, or E (Figs 3A and 3B). Group D was excluded due to crosstalk with the parent library allele *traA^F-P205A^* (Fig. 3C) [21], and the original WT group F was excluded for simplicity. Group A, represented by the lab strain DK1622, has five indels compared to the other five groups and is phylogenetically divergent (Fig. S5). Although the parent allele *traA^F-P205A^* did not recognize groups A, B, C, or E (Fig. 3C), across the four screens, positive flares were readily found from which single colonies were isolated (Fig. 3C). From the four screens, 44 clones were retained. In addition, 18 random recipient clones screened against a donor pool expressing *traA* A–F alleles were retained.

To determine the specificity of recognition, these 62 clones were individually tested against TraA recognition groups A through F in stimulation assays. Distinct recognition profiles were observed across different groups (Fig. 3D). Additionally, the sequence relationships of the five B–F parent groups from the 57 natural alleles used for library design are shown (Fig. 3E). Our findings revealed that the majority of TraA variants exhibited promiscuous interactions. In fact, only 11 of the 62 clones showed exclusive specificity to one recognition group. In contrast, 21 clones recognized four or five different recognition groups. For screens against groups B, C, and E, the purified single colonies isolated from stimulated flares of cells did not always result in a positive clone to the donor TraA group it was isolated from but nevertheless recognized one or more of the other five TraA groups. These unexpected outcomes may have occurred by serial cell–cell OME of donor motility proteins among library variants, which allowed recipients incompatible with donors to co-exist in flares with compatible recipients [14, 16]. Overall, we conclude that the residues selected for library substitutions play key roles in recognition specificity and that such changes often lead to promiscuous heterotypic interactions between TraA receptors.

These 62 positive clones were sequenced, and the amino acid biases at each of the 11 positions were visualized with WebLogos [37] (Fig. S6A). Compared to the initial library composition, several positions showed greater than two-fold enrichments in groups A, B, and C. For instance, group A-positive clones exclusively contained a serine at position 10 in all 18 positive clones. In contrast, groups D, E, and F exhibited negligible preferences compared to the original library composition. At the control residue, position 5, there was no significant change in amino acid composition or enrichment against any of the recognition groups. From this analysis, we identified positions and amino acids that showed various degrees of importance in recognition specificity. Additionally, residues absent from WebLogos may reflect detrimental specificity residues toward those recognition groups.

To help determine which positions were important for recognition, the fold change of preferred amino acids was calculated (Fig. S6B and C). Greater than two-fold enrichments were observed at the same six positions in groups A and C, five in group B, and no major enrichments were found in groups D, E, and F. These findings correlated with the phylogenetic relationships or distance among these six parent alleles (Fig. S5B), where closely related alleles to the library showed little or no preference for residues that change specificity. Additionally, crosstalk among receptors was expected to be more likely among closely related proteins [38], a trend generally found (Fig. 3D). Finally, since receptors from groups A, C, and B were more distantly related to the F^P205A^ library than groups D, E, and F, the former exhibited a greater degree of amino acid substitutions biases than the latter.

### Homotypic to heterotypic recognition conversion

**Figure 4:**
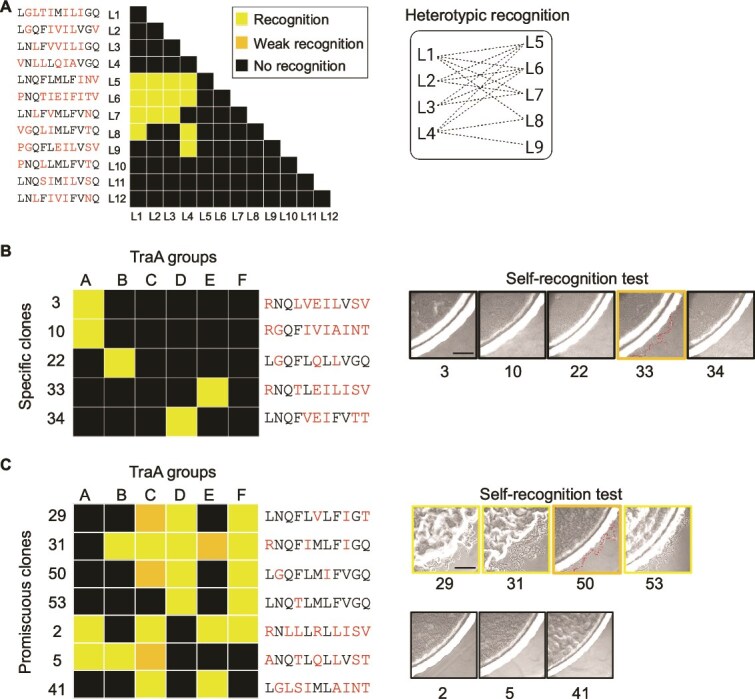
Ability of TraA variants to recognize self by homotypic binding. (A) Twelve functional variants active against the library, but not against groups A–F (from Fig. 2), exhibit no homotypic recognition. (B) TraA variants that only recognize one group among A–F (numbers refer to rows/clones from Fig. 3D). Right panel, stimulation assays between donor and recipients with same *traA* allele. Dotted red lines mark swarm fronts with weak stimulation. (C) TraA promiscuous variants tested as in B. Tables: yellow, stimulation; shaded yellow, weak stimulation; black, no stimulation. Red letters substituted amino acids from library. Scale bars, 200 μm.

In nature, recognition occurs only via homotypic binding between identical TraA receptors. This helps to ensure clonality, partially through the exchange of polymorphic toxins. In contrast, our screen frequently identified alleles involved in heterotypic allele recognition. To test for self (homotypic) recognition, we selected 12 of the 50 random isolates that were incompatible with groups A–F but nevertheless were functional when screened against the library (Fig. 2), where the same alleles were placed in donor and recipient strains for stimulation assays. Strikingly, none of these 12 variants recognized self, but nine exhibited heterotypic recognition within this group, where eight were also promiscuous (Fig. 4A). In addition, two alleles with different sequences exhibited the same recognition profiles, indicating functional degeneracy within the interfacial residues. Next, we selected an additional 12 clones from our characterized 62 clones (Fig. 3D). Here, five selected clones recognized only one of the A–F groups, while the other seven were promiscuous. For the promiscuous clones, four of seven recognized self to varying degrees, while only one of the five selective clones also exhibited weak self-recognition (Fig. 4B and C). Overall, we conclude that heterotypic interactions were more likely than homotypic recognition based on screening library clones.

### Distant TraA alleles were not library compatible

**Figure 5:**
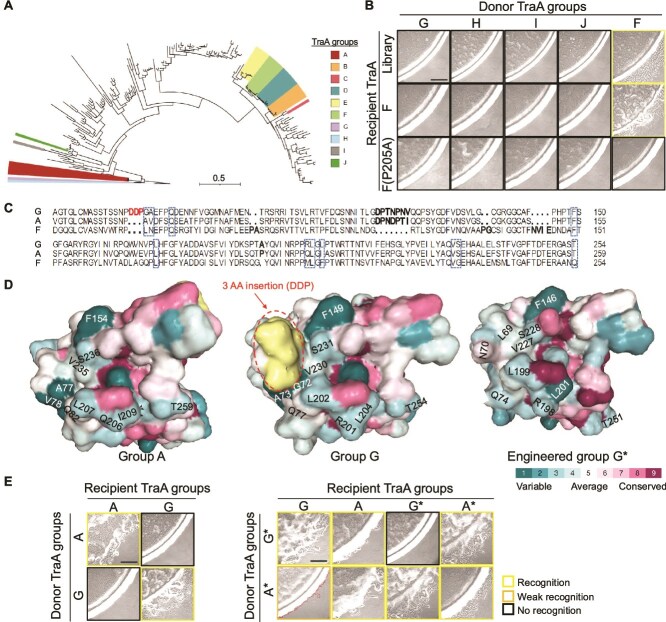
Rational design of TraA receptors. (A) ML tree of VDs from 193 Myxococcaceae TraA orthologs, including sequences with indels. TraA groups A–J are color-coded. Scale bar represents the number of substitutions per amino acid site. (B) Library screen against TraA groups G–J and F as a control. (C) VD sequence alignment highlighting indels between groups A, F, and G. The 11 positions used in library design are boxed, including the control residue. (D) AlphaFold predicted structural differences in the VD region among TraA groups A, G, and G^*^. The 10 library positions are marked. Residues are colored by the degree of conservation. (E) Stimulation outcomes of engineered variants TraA-A^*^, TraA-G^*^, and their parent alleles. Dotted lines mark weak stimulation swarm fronts. Scale bars, 200 μm.

Given the high frequency of identifying library variants that recognize groups A through F, we expanded the screen against distant TraA recognition groups G through J, which originated from non *Myxococcus* genera (Fig. 5A) [33]. Using the same sensitive stimulation assay, we were unable to identify positive clones from our library (~32 400 variants) against these four divergent TraA receptors (Fig. 5B). We conclude that these alleles, which contain various numbers of indels (Fig. S5A), were too divergent to interact with our *traA^F-P205A^*-based library.

### Engineered TraA receptors

It was notable that library variants were isolated against group A, which contains five VD indels compared to the library and was phylogenetically distant (Fig. 5A), but we were unable to identify positive clones from groups G through J, which had a similar number of indels (Fig. S5A). This raised the question: how did the library recognize group A, but not these other groups?

To investigate the molecular basis of library incompatibility, we focused on sequence and structural differences between group A and G. Representative alleles from these groups were similar in that they both shared five indels compared to groups B–F (Figs 5C and S5A). Comparing the A and G sequences, a single three-amino acid indel existed (Fig. 5C). According to AlphaFold predictions, these three residues formed an acid bulge on the interface surface (Fig. 5D). Given that library variants recognized group A, and that these three amino acids insertion represented a notable difference between groups A and G, we hypothesized this bulge played a discriminating role preventing promiscuous TraA library interactions. To test this, we constructed an in-frame deletion that removed these amino acids (D69, D70, and P71; Fig. 5C) and substituted the two adjacent interface residues with those in the *traA^F-P205A^* library (DDPGA → LN). Strikingly, this mutant (G^*^) displayed a relaxed specificity toward group A, and it retained its ability to recognize the parent G receptor (Fig. 5E). However, the G^*^ mutant did not recognize self, thus it transitioned from homotypic to heterotypic recognition. Additionally, we constructed a reciprocal allele in group A (adding DDP before AV, named A^*^), which showed homotypic and heterotypic recognition with A^*^, A, G, and G^*^. Therefore, these variants exhibited promiscuous interactions compared to their WT alleles.

### Cell–cell adhesion and OME visualization

**Figure 6:**
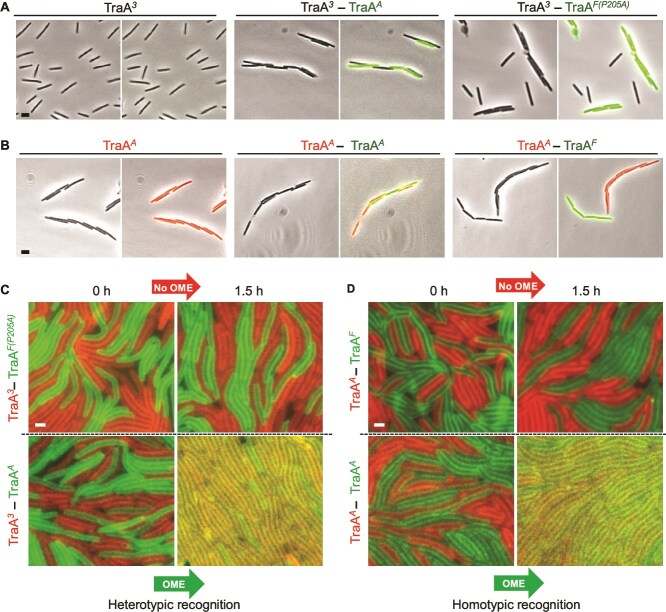
Homotypic to heterotypic switch for cell–cell adhesion and OME. (A) Library variant *traA^3^* cultured alone (no reporter), with heterotypic *traA^A^* (sfGFP) partner, or with *traA^F-P205A^* (sfGFP) parent library variant. (B) *traA^A^* strain (mCherry) cultured alone, cocultured with *traA^A^* (sfGFP) or *traA^F^* (sfGFP) strains. Superscript letters designate recognition groups. Scale bar, 2 μm. (C) TraA heterotypic recognition directs OME by library variant. *traA^3^* (mCherry) mixed with *traA^F-P205A^* (sfGFP), or with *traA^A^* (sfGFP). For noncompatible TraA receptors, no OME (top panels), whereas compatible OME receptors transform cells into phenotypically homogeneous tissue-like populations (yellow/orange) (bottom right panels). Bar, 1 μm. (D) Among WT receptors, only TraA homotypic recognition mediates OME after indicated incubations. *traA^A^* (mCherry) mixed with *traA^F^* (sfGFP), or with *traA^A^* (sfGFP). See Supplementary Figs S7 and S8 for single channel micrographs and Supplementary Table S1 for strain details. Scale bar, 1 μm.

TraA homotypic recognition promotes cell–cell adhesion upon receptor overexpression [13, 21, 33]. We tested whether heterotypic recognition also results in cell–cell adhesion. The library *traA^3^* variant, which only recognized the group A allele (Figs 3D and 4B), was monocultured or cocultured with a *traA^F-P205A^* strain labeled with sfGFP. Here *traA^3^* cells did not bind to themselves, nor to the parent *traA^F-P205A^* strain, but instead bound heterotypically with *traA^A^* (group A) cells labeled with sfGFP (Fig. 6A), as implied by the stimulation results (Figs 3D and 4B). These results contrast with WT TraA homotypic binding, where cell–cell adhesion occurs only between cells with identical TraA receptors (Fig. 6B) [21].

TraA recognition leads to the exchange of OM goods [33], and here we sought to directly visualize heterotypic OME. To do so, cells were labeled with SS_OM_-GFP or SS_OM_-mCherry lipoprotein reporters that are transferred by OME [39]. Initially, following mixing and plating, cells were phenotypically distinct (red or green). Strikingly, *traA^A^* and library-derived *traA^3^* cells became double-labeled and were phenotypically indistinguishable after 90 min (Fig. 6C). In contrast, no OME occurred between cells expressing *traA^3^* and *traA^F-P205A^*. As controls, OME occurred between cells expressing identical WT TraA receptors but not between cells expressing the WT group A and F alleles (Fig. 6D). Together, these findings confirm that TraA variants switched binding partners from homotypic to heterotypic.

## Discussion

The specificity of natural OME is mediated by homotypic interactions between TraA cell surface receptors, which enables neighboring cells to distinguish self from nonself. This recognition, in turn, leads to beneficial or harmful outcomes. These properties of the *traAB* locus represent hallmark features of “greenbeard” social genes, which have unique abilities to identify other individuals with the same allele to confer beneficial or spiteful behaviors [6, 40, 41]. Consistent with this role, the polymorphisms in TraA provide the basis for specificity where, as shown here, the protein is amenable to amino acid substitutions and indels, providing an evolutionary platform for diversification. Although previous mutagenesis studies showed that TraA specificity was changed by swapping VD domains or even by single amino acid substitutions at a key recognition switch residue [21], the molecular basis of dozens—if not hundreds—of different specific homotypic recognition groups remained unknown. Here, we used sequence alignments and structure-based modeling to predict key residues involved in interfacial specificity. We then tested these residues with a targeted combinatorial library. Strikingly, we found that sequence variations in particular TraA residues frequently generate promiscuous variants that interact with multiple WT recognition groups. In contrast, promiscuous TraA receptors are not observed in nature, indicating other selective forces are at play that favor specific homotypic binding [42].

Our rational approach effectively identified specificity residues involved in protein–protein interactions. Among the 11 positions queried, a subset of six positions played key roles dictating specificity within TraA groups A, B, and C (Fig. S6), suggesting that for these groups, the other residues are less important for specificity. In contrast, among groups D, E, and F (WT allele), no position stood out for changing specificity (Fig. S6). One explanation for the latter is that these groups are closely related to themselves and to the *traA^F-P205A^* library (Fig. 3E), thus any number of substitutions can change specificity, and no position was overtly important. Consistent with this, of the 18 random clones tested against groups A–F (Fig. 3D, bottom clones), groups D, E, and F showed the highest frequency of positive clones, 12, 10, and 17, respectively, while the positive clone frequency against groups A, B, and C was much lower, 0, 5, and 8, respectively.

In addition to substitutions, insertions and deletions can change specificity [43–45]. Here, the natural TraA^G^ allele contains a three-amino-acid insertion that results in a charged steric bulge on the recognition interface. Our data indicate that this insertion acts as a “negative constraint” that discriminates against nonspecific TraA interactions [1]. We tested this with a TraA^G^*^^ in-frame deletion variant (DDPGA → LN) and indeed found it lost specificity and became promiscuous. Thus, this acid bulge serves as a negative constraint against promiscuous binding. Collectively, our findings open the door for engineering new TraA recognition receptors, where engineering negative constraints are critical to ensure homotypic specificity. These lessons are also applicable to engineering specificity in other protein–protein interactions.

Our experimental findings provide tangible insights into the evolution of homotypic and heterophilic receptors. First, homophilic receptors allow a comparatively simple evolutionary path because a single protein is involved in recognizing an identical partner on another cell. This contrasts with heterophilic receptors, which must bind distinct partners, and therefore requires coordinated, reciprocal evolution to direct binding specificity. For homophilic receptors, gene duplication followed by mutational diversification can generate new specificities without disrupting recognition, because one of the paralogs retains self-binding ability. This evolutionary mechanism explains how families of proteins involved in heterophilic binding, such as nectins and cadherins, evolved through duplication and diversification [46, 47]. Similarly, homophilic families can evolve by gene duplication and diversification, as found for protocadherins in mammals and Dscam isoforms in *Drosophila*, where their extreme isoform diversity is used for neuronal self-avoidance and proper circuit assembly [48–50]. Our findings with TraA show that sequence diversification can readily form alleles with altered specificities, which in turn can be acted upon by selective forces to evolve new pathways with increased fitness.

Similar promiscuity is observed in heterotypic nonself-recognition systems, including those in Solanaceae plants and Basidiomycete fungi. In Solanaceae, self-incompatibility (SI) prevents self-fertilization and enforces outcrossing. This process is controlled by a highly polymorphic S-locus, where multiple S-loci F-box (SLF) proteins in pollen collectively recognize and detoxify a suite of nonself S-RNases expressed in the pistil [51, 52]. Promiscuous molecular recognition plays a role in the evolution of RNase-based SI by maintaining a dynamic balance between the gain and loss of specificity, thereby increasing the evolvability of the recognition network [52]. Similarly, in Basidiomycete, mating compatibility is determined by multiallelic pheromone–receptor systems and homeodomain transcription factors, where receptor–ligand interactions can exhibit limited promiscuity and tolerate cross-reactivity, enabling recognition among diverse nonself partners [53, 54]. Together, these observations support a unifying model in which molecular promiscuity provides a flexible evolutionary path for expanding recognition repertoires across the domains of life.

### Working model for the evolution of TraA homotypic specificity

Our screen yielded two unexpected findings. The first was the high frequency of clones with altered specificities. This shows that TraA has a malleable VD scaffold that is amenable to specificity changes through mutations. Given this, in future *traA* libraries, we would design a library with an average of one or two substitutions per clone instead of five used here. However, having said this, when screening for altered recognition to divergent alleles, e.g. groups G-J, a high number of substitutions and indel changes may be a more advantageous approach. Second, given that WT TraA receptors function in homophilic binding, we were struck by the low frequency of library variants that act in homophilic recognition. For example, from a random selection of 12 library clones, none recognized self, though they functioned in heterotypic recognition (Fig. 4A). Since heterotypic and promiscuous binders were easily found, while natural TraA alleles are homotypic specific, natural selective forces must drive selectivity toward the latter.

**Figure 7:**
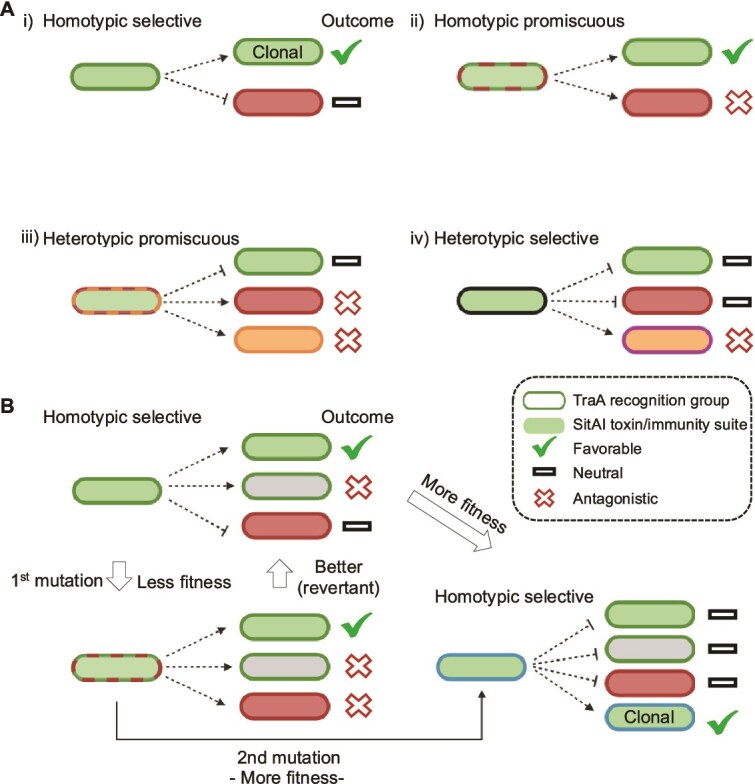
Working models of selective conditions that promote TraA homotypic recognition. (A) Four types of TraA recognitions that occur during evolutionary diversification. Distinct TraA recognition groups are designated by outlined cell colors; hatched outlines designate promiscuous recognition. Cytoplasmic color designates different SitAI toxin/immunity suites. (B) Model illustrating the evolutionary steps to create a new homotypic TraA–TraA recognition specificity through promiscuous intermediate. See text for details.

OME shares characteristics of “Red Queen dynamics,” where *traA* alleles continuously diversify to avoid exchanging polymorphic toxins with nonself [55]. In this evolutionary arms race between the TraA receptor and *sitAI* selfish mobile elements, changes in one component drive reciprocal counter-selection in the other, fueling cycles of innovation to maintain fitness. As shown in prior work [15, 20], population dynamics is driven by HGT of polymorphic toxin-immunity cassettes, which allows recent recipients to kill siblings with compatible TraA receptors. Here, we provide a rationale for the evolution and maintenance of TraA polymorphisms while maintaining homotypic specificity (Fig. 7A), which contrasts with our experimental findings. We suggest that during TraA diversification, sequence variants fall into four functional categories: homotypic selective, heterotypic selective, heterotypic promiscuous, and homotypic promiscuous. Homotypic selective represents the WT state, which enhances population fitness by promoting cooperation among self while remaining neutral toward nonself cells with divergent receptors. Homotypic promiscuous variants support self-cooperation but also result in antagonism or harm with nonself due to OME of SitA toxins [14, 16, 56]. Heterotypic selective is limited to specific strains with a different *traA* allele, fostering targeted OME-mediated antagonism. In contrast, heterotypic promiscuous recognition is likely the most detrimental, as TraA fails to recognize self, while promoting OME and antagonism with nonkin. We propose that these promiscuous interactions—frequently found in our screen—represent intermediate states toward homotypic specificity, which rarely occurs by random mutations.

We propose that new TraA specificities typically arise through the generation of promiscuous variants, driven by a reward-and-punishment system arising from cell–cell interactions. These steps will lead to social diversification that may ultimately enhance overall population fitness and thus favored by natural selection (Fig. 7B) [20]. In wild myxobacteria populations, homotypic selective recognition produces three outcomes: promoting cooperation among self or kin, antagonizing individuals with compatible TraA receptors but different SitA toxins, and maintaining neutrality toward nonkin with divergent TraA groups.

TraA diversification likely proceeds through a series of changes, where a newly established *traA* allele, formed by a mutation or HGT and recombination [15, 17], broadens TraA recognition. In some cases, this will initially be advantageous by avoiding OME with antagonistic cells that have compatible TraA receptors. However, over a long duration increased promiscuity will increase the frequency of antagonism due to diverse sets of SitA toxin repertoires. Therefore, subsequent *traA* mutation(s) are selected that narrows specificity toward nonantagonistic self, resulting in novel homotypic recognition that promotes clonal cooperation while remaining neutral toward others (Fig. 7B). By generating a new recognition specificity, TraA stabilizes cooperation by avoiding promiscuous and antagonistic interactions with nonself. Future work seeks to test this model by using genetic screens that select against promiscuous TraA recognition, while simultaneously selecting for beneficial homotypic OME among kin. In sum, our work provides a basis for future experiments to probe the mechanisms and dynamics of cell–cell recognition, a process that underlies the important transitions in the evolution of multicellularity.

## Supplementary Material

Supplementary_material_final_wrag102

## Data Availability

All data are present in the main text or the supplementary materials.
