# Supplementary material for: Engineered kin recognition specificities in the TraA cell surface receptor
Source: ISME J. 2026 Apr 23;20(1):wrag102. doi: 10.1093/ismejo/wrag102 (PMC13184517; doi:10.1093/ismejo/wrag102)
Supplement: Supplementary_material_final_wrag102 [file supplementary_material_final_wrag102.docx]

Supplemental information

**Engineered kin recognition specificities in the TraA cell surface receptor**

Tingting Guo^1^ and Daniel Wall^1*^

^*^ Corresponding author. Email: dwall2@uwyo.edu.

Supplemental Figures and Tables

**
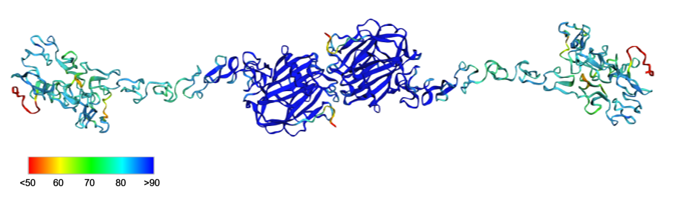
**

**Fig. S1. Backbone representation of TraA-TraA homotypic binding.** Structure predicted by AlphaFold2 and color-coded by pLDDT confidence scores.


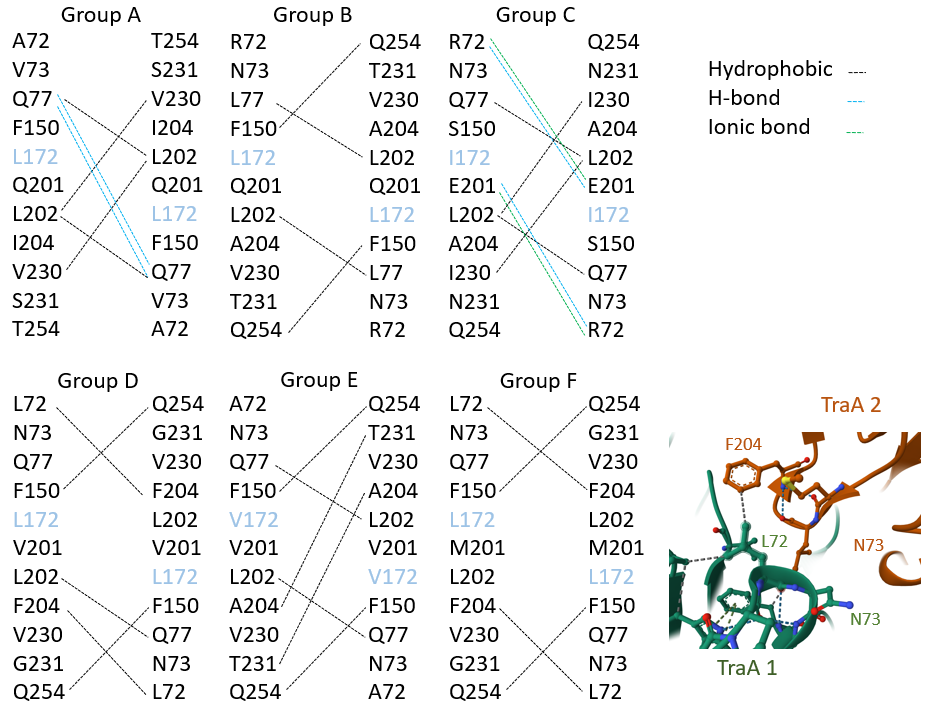


**Fig. S2. AlphaFold2 homodimer structure predictions and interacting interfacial residues between TraA monomers.** Noncovalent interfacial bonds were visualized in RCSB PDB ([www.rcsb.org](http://www.rcsb.org)) [1]. Predicted bonds shown are for recognition groups A-F where the represented alleles are from strains DK1622, DK816, Pali, A96, DK805 and HW-1, respectively. Residues numbers are based on the group F sequence (HW-1) as shown in Fig. 1. The sequence numbers are inverted to represent the ~180° orientation between monomers in the dimer structure. For library design only groups B-F were considered because group A has a divergent sequence from other five groups that includes indels. Bottom right panel shows representative predicted bonds between TraA monomers from the group F (HW-1) structure. Here, a predicted hydrophobic bond between L72 and F204 monomers is shown. Although N73 does not form a bond here, or in the six represented groups, it is located at the dimer interface and in other sequences is predicted to form noncovalent bonds.


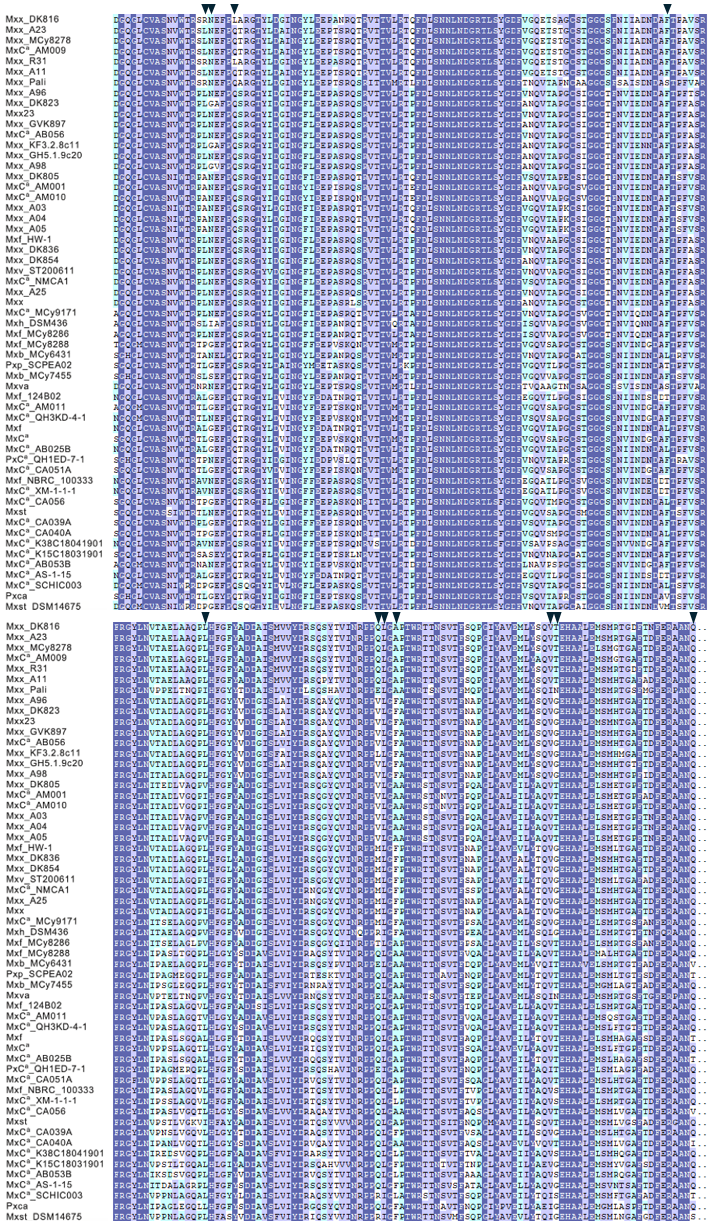


**Fig. S3. Sequence alignment of the TraA VD from groups B to F across 57 sequences that contain no indels.** Black triangles, 11 positions substituted for library construction. Homologies: Blue, 100%; violet, >75%; cyan >33%.


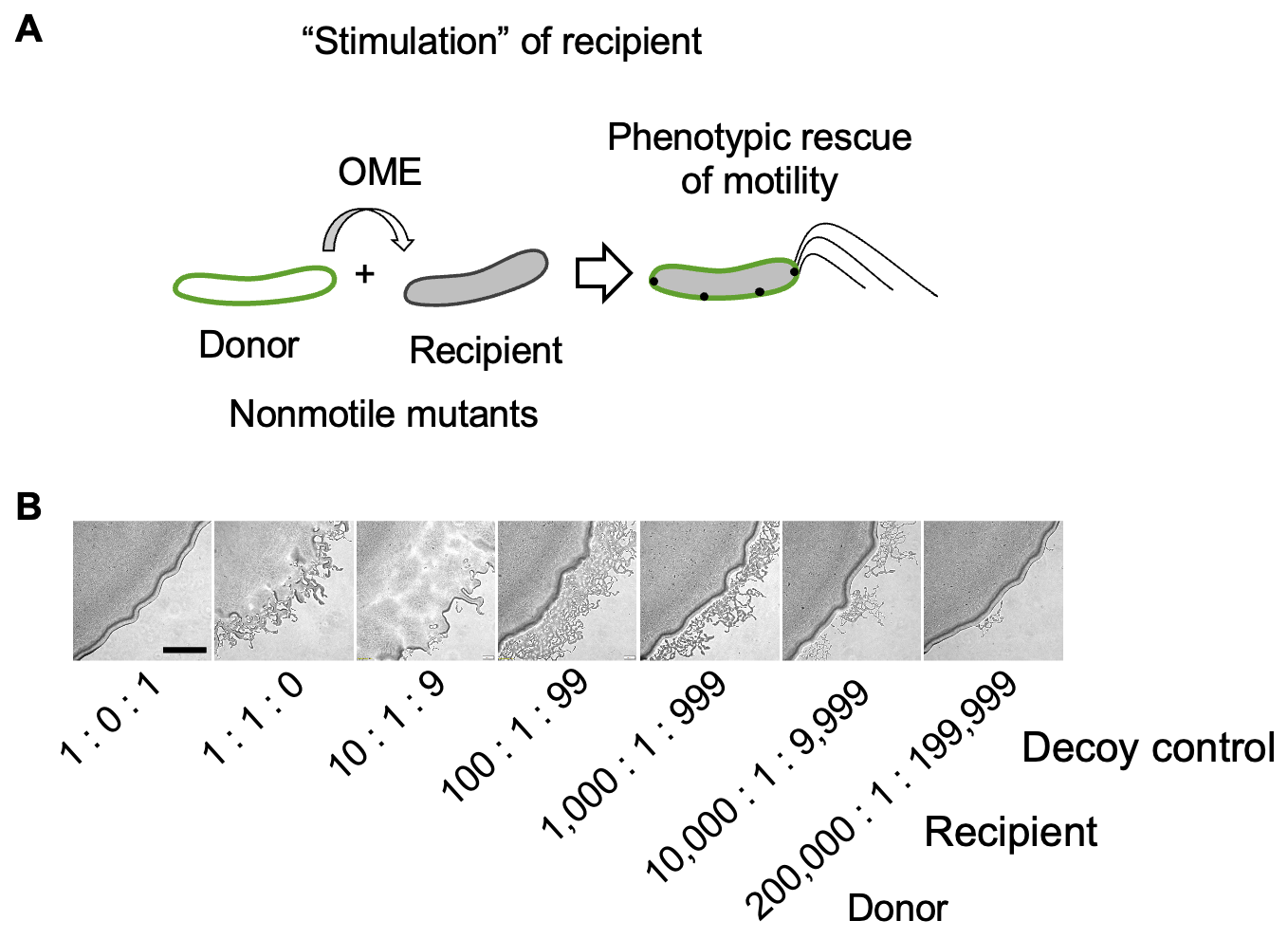


**Fig. S4.** **Outline and sensitivity of library screen.** **A**) Schematic representation of the stimulation assay. Recipient contains two mutations in outer membrane motility lipoproteins (Δ*cglC* Δ*tgl*) required for A- and S-motility, respectively. The nonmotile donor strain contains these WT proteins (green) and transfers them by OME to transiently restore S-motility (type IV pili) and A-motility (black dots, focal adhesions) to the recipient. **B**) Stimulation assay sensitivity. Donor strain (*traA*^DK805^) was mixed with the stimulatable recipient strain *traA*^DK805^, and the non-stimulatable strain *traA*^MCy8401^ at indicated ratios, to maintain the donor ratio of one half of total cells. See Table S1 for strain details. Micrographs at 24 h; scale bar, 200 µm.

**
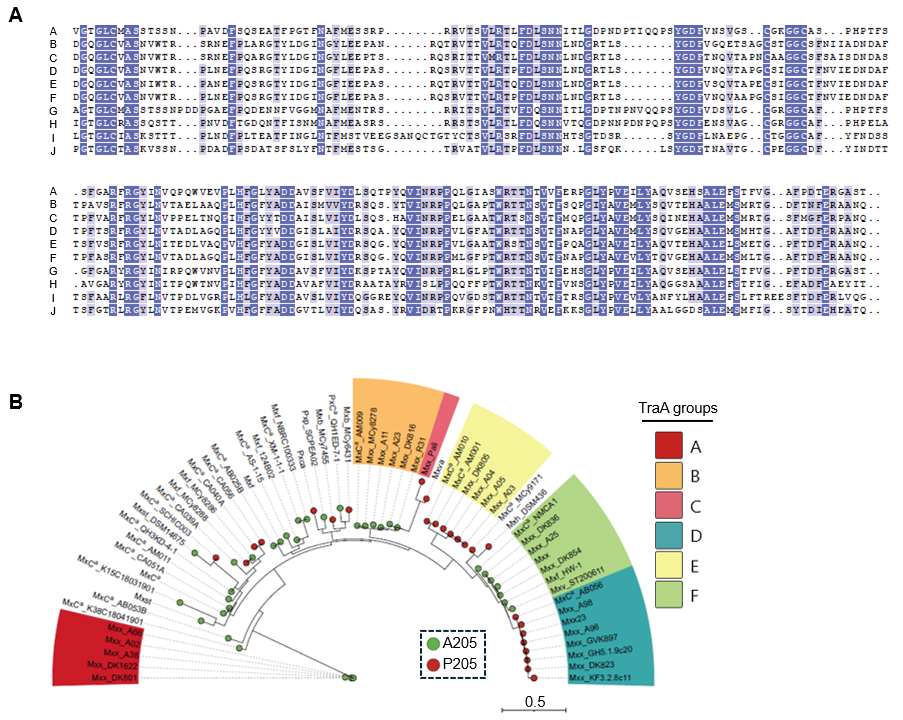
**

**Fig. S5. A**) Sequence alignment of the VD region from representative TraA group A through J members. Recognition groups labeled on the left. Homologies: Blue, 100%; violet, >75%. **B**) Maximum likelihood tree of the VDs from 62 *Myxococcaceae* TraA orthologs that includes sequences with indels. TraA groups A–F color-coded. Scale bar, number of substitutions per amino acid position.


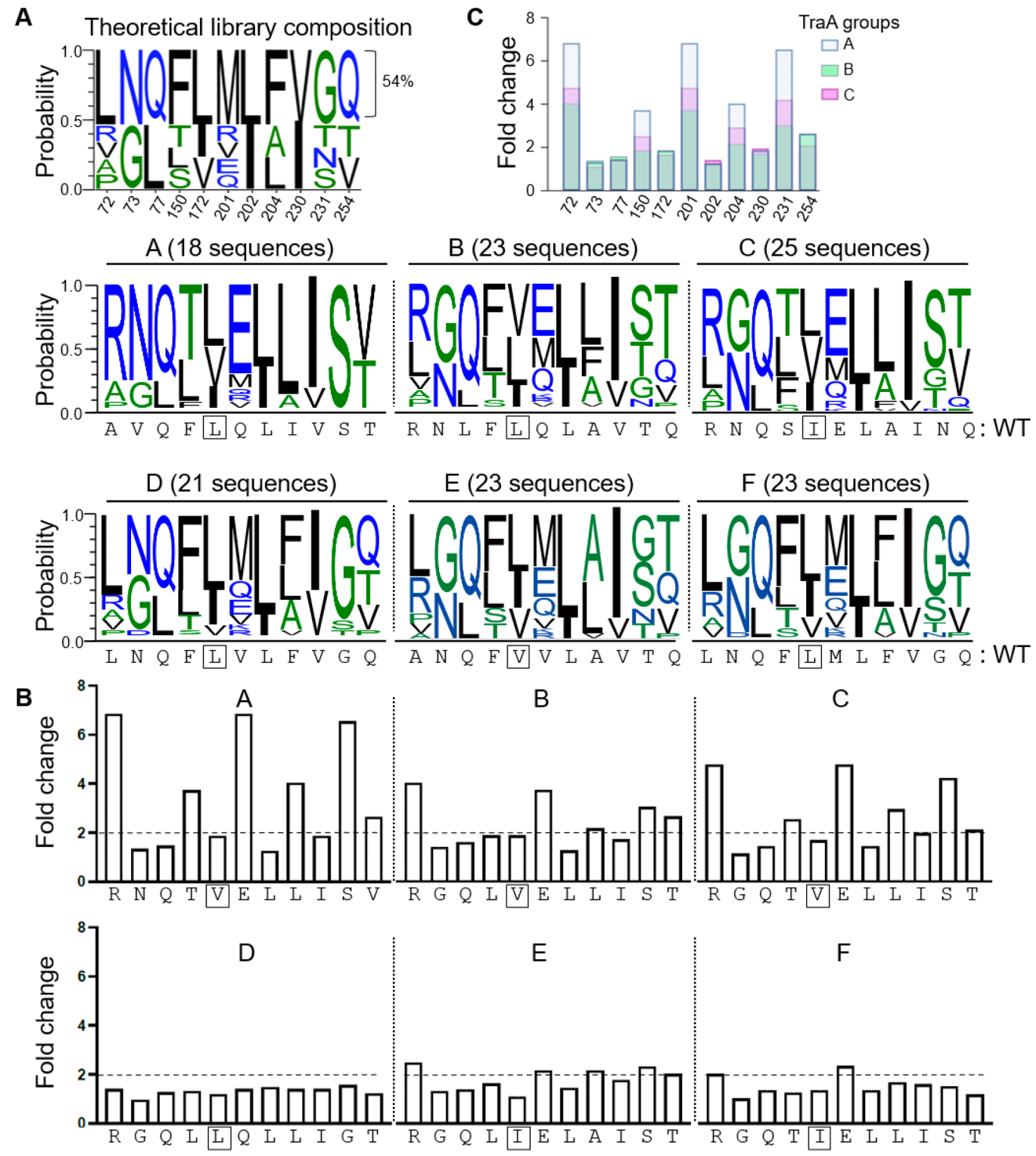


**Fig. S6. Amino acid enrichments from different TraA recognition group screens.** **A**) WebLogos of amino acid preferences from TraA positive clones against groups A to F. The starting library bias also shown (top left). Residues color-coded: hydrophilic (blue), neutral (green), and hydrophobic (black). Control position 5 boxed. **B**) Amino acid fold-change enrichments shown (bottom of graphs). Enrichments >2-fold shown (dashed lines). **C**) Fold-change highlighting amino acid enrichments in groups A, B and C.


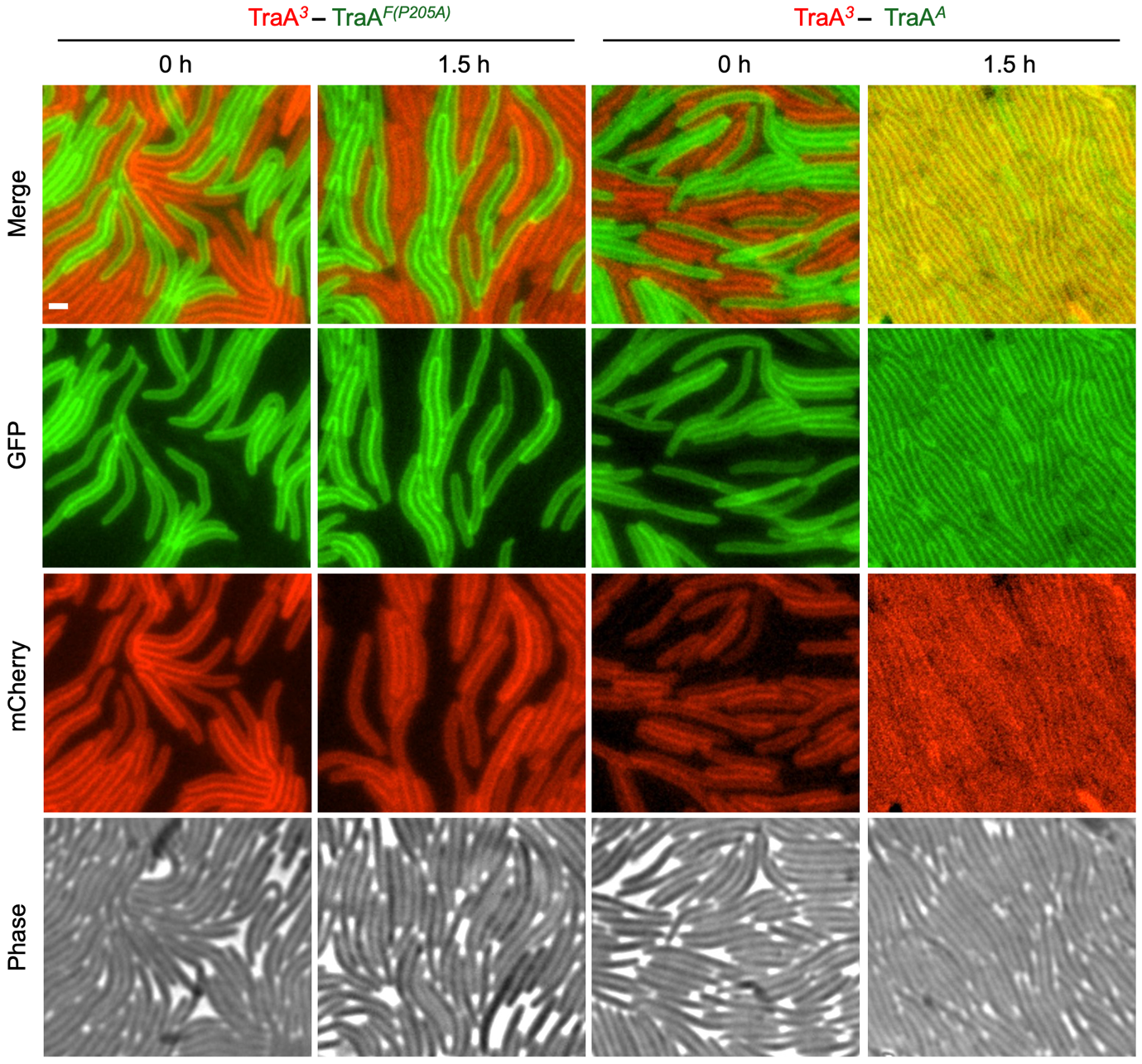


**Fig. S7.** **Homotypic TraA-dependent OME between cells harboring transferable SS_OM_-GFP or SS_OM_-mCherry reporters.** Complementary to Fig. 6C, showing single channel images with merged images. Scale bar, 1 μm.

**
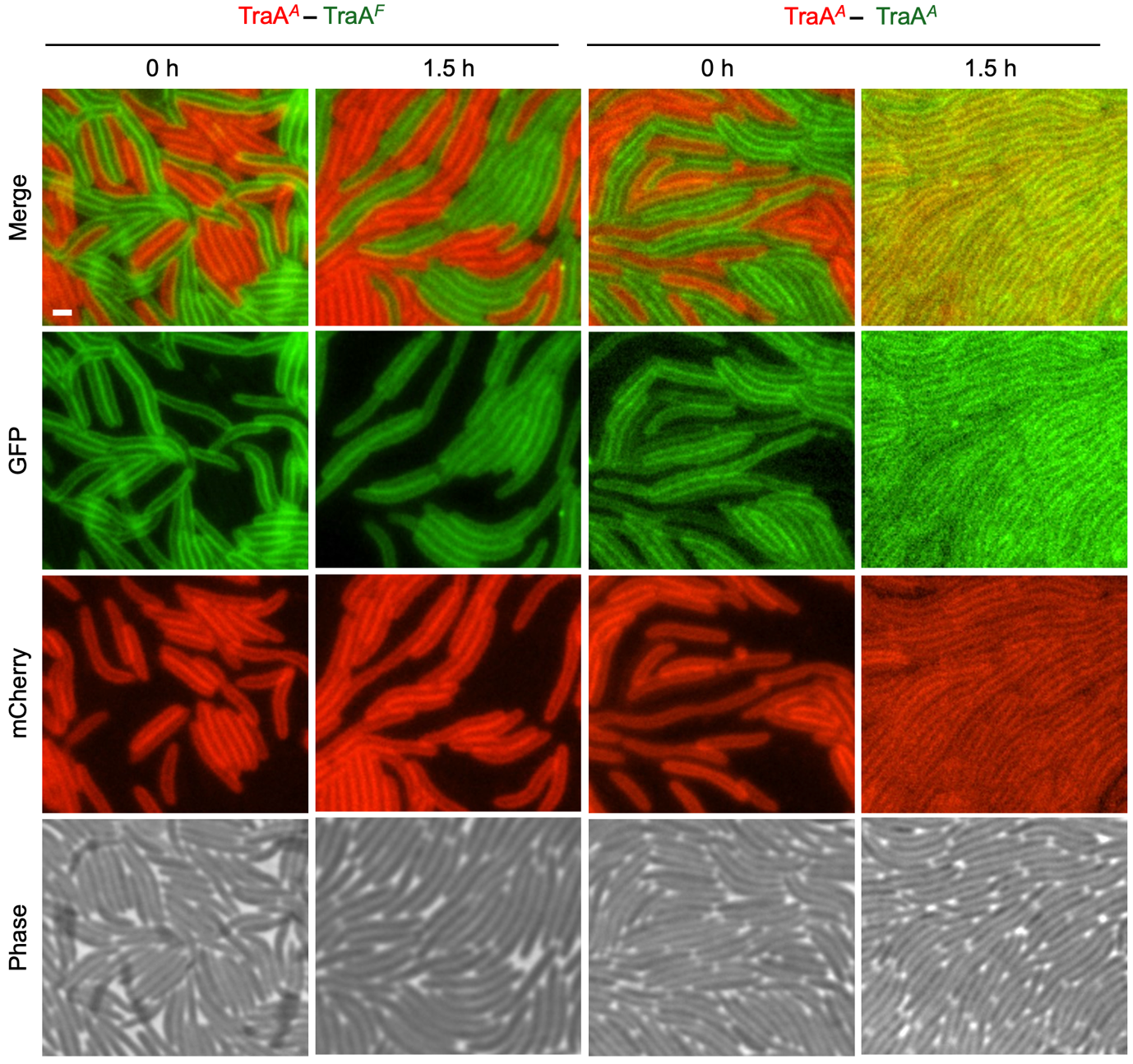
**

**Fig. S8. Heterotypic TraA-dependent OME between cells harboring transferable SS_OM_-GFP or SS_OM_-mCherry reporters.** Complementary to Fig. 6D, showing single channel images with merged images. Scale bar, 1 μm.

**Table S1** **Plasmids and strains used in this study**

| **Plasmids** | **Relevant features** | | **Source** |
| --- | --- | --- | --- |
| pMR3487 | IPTG-inducible promoter, Tc^R^ |  | [2] |
| pΔ*cglC* | Δ*cglC* (deletion cassette) in pBJ114, *galK*, Km^R^ |  | Lotte Søgaard-Andersen |
| pDP28 | Δ*traA* (deletion cassette) in pBJ114, *galK*, Km^R^ |  | [3] |
| pPC16 | P*_pilA_*-RBSsyn-*traA*^DK805^ in pDP22 (Mx8 *attP*), Km^R^ |  | [4] |
| pPC4 | P*_pilA_*-RBSsyn-*traAB*^Mf^ in pDP22, Km^R^ |  | [4] |
| pPC5 | P*_pilA_*-*traA*^DK816^*traB*^DK1622^ in pDP22, Km^R^ |  | [4] |
| pPC26 | P*_pilA_*-RBSsyn-*traA*^MCy5730^ in pDP22, Km^R^ |  | [5] |
| pDP27 | P*_pilA_*-RBSsyn-*traA*^DK1622^ in pDP22, Km^R^ |  | [3] |
| pXW6 | P*_pilA_*-*SS_OM_*-*mCherry* in pKSAT, Sm^R^ |  | [6] |
| pPC43 | P*_pilA_*-*SS_OM_*-*gfp* in pKSAT, Sm^R^ |  | [5] |
| pPC1 | P*_pilA_*-*SS_OM_*-sfGFP in pKSAT, Sm^R^ |  | [4] |
| pDP21 | P*_pilA_*-*traA*^DK1622^ in pDP22, Km^R^ |  | [7] |
| pDP22 | pSWU19 Mx8 *attP* cassette, P*pilA*, Km^R^ |  | [3] |
| pDP23 | P*_pilA_*-*traA*^DK816^ in pDP22, Km^R^ |  | [3] |
| pDP26 | P*_pilA_*-*traA*^Pali^ in pDP22, Km^R^ |  | [3] |
| pDP24 | P*_pilA_*-*traA*^A96^ in pDP22, Km^R^ |  | [3] |
| pDP25 | P*_pilA_*-*traA*^Mf^ in pDP22, Km^R^ |  | [3] |
| pPC27 | P*_pilA_*-*traA*^MCy8401^ in pDP22, Km^R^ |  | [5] |
| pPC28 | P*_pilA_*-*traA*^And48^ in pDP22, Km^R^ |  | [5] |
| pPC36 | P*_pilA_*-*traA*^MCy8337/DK1622^ in pDP22, Km^R^ |  | [5] |
| pXW7 | P*_pilA_*-*traB*,pCR-XL-TOPO; Δ*neoR/kanR*,Mx9 *attP* Zeo^R^ |  | [5] |
| pPC61 | P*_pilA_*-*traA*^Mf(P205A)^-pSWU19 |  | Lab collection |
| pPC62 | P*_pilA_*-*traA*^Mf(P205A)^-*traB*-pSWU19 |  | Lab collection |
| pTG2909 | pMR3487-*traAB*^Mf^, Tc^R^ |  | This study |
| pTG2910 | P*_pilA_*-RBSsyn-*traA*^MCy5730 (DDPGA→LN)^ in pDP22, Km^R^ |  | This study |
| pTG2911 | P*_pilA_*-RBSsyn-*traA*^DK1622^ *^(^*^add DDP before AV^*^)^* in pDP22, Km^R^ |  | This study |
| **Strains** | **Relevant features** | **Experimental use** | **Source** |
| Top10 | *E. coli* cloning strain | Cloning | Lab collection |
| DH5α | *E. coli* cloning strain | Cloning | Lab collection |
| DK1217 | *M. xanthus aglB1*(*aglQ1*), A^ꟷ^S^+^, parent to DK1622 |  | [8] |
| DK1622 | WT *M. xanthus*, motile (A^+^S^+^) |  | [8] |
| DK8601 | DK1617 *aglB1* (*aglQ1*) Δ*pilA*::Tc, nonmotile, Tc^R^ | Fig. 3C and 5E | [9] |
| DW1467 | DK8601 Δ*traA* (markerless), Tc^R^ |  | [3] |
| DK6204 | DK1622 Δ*mglBA* (markerless), nonmotile |  | [10] |
| DK8615 | DK1622 Δ*pilQ* (markerless) |  | [11] |
| DW1466 | DK1622 *tgl*::Tc Δ*cglC* (markerless), nonmotile, Tc^R^ | Fig. 2C and 5E | [7] |
| DW2220 | DW1466 Δ*traA* (markerless), Tc^R^ |  | [4] |
| DK101 | *pilQ1* aka FB or DZF1 |  | [11, 12] |
| DK1253 | DK101 *tgl1* |  | Lab collection |
| DW2929 | DK1253 Δ*cglC* (markerless) |  | This study |
| DW2930 | DK1253 *tgl1* Δ*cglC* Δ*traA* (markerless) | Library strain | This study |
| DW2302 | DK6204 Δ*traA* | Library strain | Lab collection |
| DW1480 | DK1622 Δ*traA* |  | [13] |
| DW2930 | DW1480 (pXW6), Sm^R^ |  | This study |
| DW2221 | DW2220 (pDP23), Km^R^, Tc^R^ | Fig. 2C | [4] |
| DW2224 | DW2220 (pDP26), Km^R^, Tc^R^ | Fig. 2C | [4] |
| DW2222 | DW2220 (pDP24), Km^R^, Tc^R^ | Fig. 2C | [4] |
| DW2234 | DW2220 (pPC16), Km^R^, Tc^R^ | Fig. 2C | [4] |
| DW2223 | DW2220 (pDP25), Km^R^, Tc^R^ | Fig. 2C, 3C and 5B | [4] |
| DW2303 | DW2220 (pPC61), Km^R^, Tc^R^ | Fig. 3C and 5B | Lab collection |
| DW2248 | DW2220 (pPC26), Km^R^, Tc^R^ | Fig. 5E | [5] |
| DW2931 | DW2302 (pTG2910), Km^R^ | Fig. 5E | This study |
| DW2932 | DW2220 (pTG2910), Km^R^ | Fig. 5E | This study |
| DW2933 | DW2302 (pTG2911), Km^R^ | Fig. 5E | This study |
| DW2934 | DW2220 (pTG2911), Km^R^, Tc^R^ | Fig. 5E | This study |
| DW1468 | DW1467 (pDP23), Km^R^, Tc^R^ | Fig. 3C | [3] |
| DW1471 | DW1467 (pDP26), Km^R^, Tc^R^ | Fig. 3C | [3] |
| DW1469 | DW1467 (pDP24), Km^R^, Tc^R^ | Fig. 3C | [3] |
| DW2212 | DW1467 (pPC16), Km^R^, Tc^R^ | Fig. 3C | [4] |
| DW1470 | DW1467 (pDP25), Km^R^, Tc^R^ | Fig. 3C and 5B | [3] |
| DW2243 | DW1467 (pPC26), Km^R^, Tc^R^ | Fig. 5BE | [5] |
| DW2244 | DW1467 (pPC27), Km^R^, Tc^R^ | Fig. 5B | [5] |
| DW2245 | DW1467 (pPC28), Km^R^, Tc^R^ | Fig. 5B | [5] |
| DW2262 | DW1467 (pPC36, pXW7), Km^R^, Tc^R^, Zeo^R^ | Fig. 5B | [5] |
| DW2304 | DK8601 ∆*traAB* (pPC62), Km^R^, Tc^R^ |  | Lab collection |
| DW2935 | DK8615 pXW6 (pMR3487-*traA^3^*B*), Sm^R^, Tc^R^ |  | This study |
| DW1463 | DK8601 (pXW6, pDP21), Km^R^, Tc^R^, Sm^R^ | Fig. 6B | [7] |
| DW2201 | DK8601 (pPC1, pDP21), Km^R^, Tc^R^, Sm^R^ | Fig. 6, S7 and S8 | [4] |
| DW2287 | DK8601 Δ*traAB* (pPC4, pPC43), Km^R^, Tc^R^, Sm^R^ | Fig. 6B, 6D and S8 | [14] |
| DW2936 | DW2929 (pMR3487-*traA^3^B*), Tc^R^ | Fig. 6A | This study |
| DW2937 | DW2303 (pPC43), Km^R^, Tc^R^, Sm^R^ | Fig. 6A | This study |
| DW1478 | DK8615 (pXW6), Sm^R^ | Fig. 6D and S8 | [15] |
| DW2938 | DW2930 (pMR3487-*traA^3^B*), Sm^R^, Tc^R^ | Fig. 6C and S7 | This study |
| DW2939 | DW2304 (pPC43), Km^R^, Tc^R^, Sm^R^ | Fig. 6C and S7 | This study |
| DW2940 | DW2302 (pPC16), Km^R^ | Fig. S4B | This study |
| DW2234 | DW2220 (pPC16), Km^R^, Tc^R^ | Fig. S4B | [4] |
| DW2249 | DW2220 (pPC27), Km^R^, Tc^R^ | Fig. S4B | [5] |

**Table S2 Primers used in this study**

| **Primer name** | **Sequence (5’→3’)*** |
| --- | --- |
| pMR3487-*traAB*^Mf^-XbaI-F | GGATAACAATTAAGGAGGCTCTAGAATGGACGATATCCCTCATTC |
| pMR3487-*traAB*^Mf^-KpnI-R | TGATTACGAAGGCGAGCTCGGTACCCTACGGCTTGGGCGCCGAG |
| pSWU19-F | TGGTAACTGTCAGACCAAG |
| pSWU19-MX8-R | TTCGACGATGGCCTCCAC |
| pSWU19-MX8-F | ACATTGACGTGGAGGCCATC |
| 1622 add DDP before AV-R | AAGTCCACGGCCGGGTCATCCGGATTCGATGATGTGC |
| 1622 add DDP before AV-F | GATGACCCGGCCGTGGACTTCTCTCAG |
| pSWU19-R | AACTTGGTCTGACAGTTAC |
| TraA^MCy5730^-(DDPGA→LN)-R | CTGCGGGAACTCGTTGAGCGGATTCGACGAAGTGCTC |
| TraA^MCy5730^-(DDPGA→LN)-F | CTCAACGAGTTCCCGCAGGACGAA |

Restriction sites underlined.

**Table S3 Abbreviations for indicated taxa**

| **Prefix** | **Species** |
| --- | --- |
| Mxb | *Myxococcaceae bacterium* |
| MxC^a^ | *Myxococcus clade* |
| Mxf | *Myxococcus fulvus* |
| Mxh | *Myxococcus hansupus* |
| Mxst | *Myxococcus stipitatus* |
| Mxv | *Myxococcus virescens* |
| Mxva | *Myxococcus vastator* |
| Mxx | *Myxococcus xanthus* |
| PxC^a^ | *Pyxidicoccus clade* |
| PxCa | *Pyxidicoccus caerfyrddinensis* |
| Pxp | *Pyxidicoccus parkwaysis* |

^a^Unclassified species assigned to clade C.

**References**

1. Berman HM, Westbrook J, Feng Z. *et al*. The Protein Data Bank*.* *Nucleic Acids Res* 2000;**28**:235–42.
2. Iniesta Martínez AÁ, García Heras F, Abellón Ruíz J. *et al*. Two systems for conditional gene expression in *Myxococcus xanthus* inducible by isopropyl-β-D-thiogalactopyranoside or vanillate*. J Bacteriol* 2012;194:5875-85.
3. Pathak DT, Wei X, Dey A. *et al*. Molecular recognition by a polymorphic cell surface receptor governs cooperative behaviors in bacteria. *PLoS Genet* 2013;9:e1003891.
4. Cao P, Wall D. Self-identity reprogrammed by a single residue switch in a cell surface receptor of a social bacterium. *Proc Natl Acad Sci USA* 2017;114:3732–3737.
5. Cao P, Wei X, Awal RP. *et al*. A highly polymorphic receptor governs many distinct self-recognition types within the *Myxococcales* order. *mBio* 2019;10:e02751-18.
6. Wei X, Pathak DT, Wall D. Heterologous protein transfer within structured myxobacteria biofilms. *Mol Microbiol* 2011;81:315-326.
7. Pathak DT, Wei X, Bucuvalas A. *et al*. Cell contact-dependent outer membrane exchange in myxobacteria: genetic determinants and mechanism. *PLoS Genet* 2012;8:e1002626.
8. Dey A, Vassallo CN, Conklin AC. *et al*. Sibling rivalry in *Myxococcus xanthus* is mediated by kin recognition and a polyploid prophage. *J Bacteriol* 2016;198:994-1004.
9. Wall D, Kaiser D. Alignment enhances the cell-to-cell transfer of pilus phenotype. *Proc Natl Acad Sci USA* 1998;95:3054-3058.
10. Hartzell P, Kaiser D. Upstream gene of the *mgl* operon controls the level of MglA protein in *Myxococcus xanthus*. *J Bacteriol* 1991;173:7625-7635.
11. Wall D, Kolenbrander PE, Kaiser D. The *Myxococcus xanthus pilQ* (*sglA*) gene encodes a secretin homolog required for type IV pilus biogenesis, social motility, and development. *J Bacteriol* 1999;181:24-33.
12. Müller S, Willett JW, Bahr SM. *et al*. Draft genome of a type 4 pilus defective *Myxococcus xanthus* strain, DZF1. *Genome announc* 2013;1:10-1128.
13. Vassallo C, Pathak DT, Cao P. *et al*. Cell rejuvenation and social behaviors promoted by LPS exchange in myxobacteria. *Proc Natl Acad Sci* *USA* 2015;112:E2939-E2946.
14. Cao P, Wall D. Direct visualization of a molecular handshake that governs kin recognition and tissue formation in myxobacteria. *Nat Commun* 2019;10:3073.
15. Dey A, Wall D. A genetic screen in *Myxococcus xanthus* identifies mutants that uncouple outer membrane exchange from a downstream cellular response. *J Bacteriol* 2014;196:4324-4332.
